# Retraction Note: Intensification of resveratrol cytotoxicity, pro-apoptosis, oxidant potentials in human colorectal carcinoma HCT-116 cells using zein nanoparticles

**DOI:** 10.1038/s41598-025-25537-9

**Published:** 2025-10-28

**Authors:** Maan T. Khayat, Mohamed A. Zarka, Dalia Farag. A. El-Telbany, Ali M. El-Halawany, Hussam Ibrahim Kutbi, Walid F. Elkhatib, Ayman M. Noreddin, Ahdab N. Khayyat, Rania Farag A. El-Telbany, Sherif F. Hammad, Ashraf B. Abdel-Naim, Ebtesam M. Alolayan, Majid Mohammad Al-Sawahli

**Affiliations:** 1 Department of Pharmaceutical Chemistry, Faculty of Pharmacy, King Abdulaziz University, Jeddah, Saudi Arabia; 2 Department of Pharmacognosy, College of Pharmacy, The Islamic University, Najaf, Iraq; 3 Department of Pharmaceutics, Faculty of Pharmacy, Modern University for Technology and Information (MTI), Cairo, Egypt; 4 Department of Pharmacognosy, Faculty of Pharmacy, Cairo University, Cairo, Egypt; 5 Department of Pharmacy Practice, Faculty of Pharmacy, King Abdulaziz University, Jeddah, Saudi Arabia; 6 Department of Microbiology and Immunology, Faculty of Pharmacy, Ain Shams University, Cairo, Egypt; 7 Department of Microbiology and Immunology, Faculty of Pharmacy, Galala University, New Galala City, Suez, Egypt; 8 Department of Pharmacy Practice, Faculty of Pharmacy, Ahram Canadian University, 6th October City, Egypt; 9 Department of Internal Medicine, School of Medicine, University of California, Irvine, CA USA; 10 Department of Biochemistry, Faculty of Pharmacy, Modern University for Technology and Information (MTI), Cairo, Egypt; 11 Department of Pharmaceutical Chemistry, Faculty of Pharmacy, Helwan University, Cairo, Egypt; 12 PharmD Program and Basic and Applied Sciences Institute, Egypt-Japan University of Science and Technology (E-JUST), New Borg El-Arab City, Alexandria, Egypt; 13 Department of Pharmacology and Toxicology, Faculty of Pharmacy, King Abdulaziz University, Jeddah, Saudi Arabia; 14 Department of Zoology, College of Science, King Saud University, Riyadh, Saudi Arabia; 15 Department of Pharmaceutics, College of Pharmacy, The Islamic University, Najaf, Iraq; 16 Department of Pharmaceutical Technology, Faculty of Pharmacy, Kafr Elsheikh University, Kafr Elsheikh, Egypt

Retraction of: *Scientific Reports* 10.1038/s41598-022-18557-2, published online 08 September 2022

The Editors have retracted this Article. After publication, concerns were raised regarding the authenticity of the cell cycle data presented in Figures 6a through 6d and Figures 7a through 7d. Furthermore, Figures 6c and 7c appear to overlap with in Figures 6c and 7c in^1^, respectively. The Authors provided some of the original data to the Editors, but these were insufficient to address the concerns. The Editors no longer have confidence in the data underlying this Article.

Majid Mohammad Al-Sawahli, Maan T. Khayat, Dalia Farag. A. El-Telbany, Rania Farag A. El-Telbany, Mohamed A. Zarka, Ahdab N. Khayyat, Hussam Ibrahim Kutbi, Ashraf B. Abdel-Naim, and Ebtesam M. Alolayan disagree with this retraction. Ali M. El-Halawany, Walid F. Elkhatib, Ayman M. Noreddin, and Sherif F. Hammad have not replied to correspondence from the Editors about this retraction.
